# Hyperpolarized long-lived nuclear spin states in monodeuterated methyl groups†
†Electronic supplementary information (ESI) available. See DOI: 10.1039/c8cp00253c


**DOI:** 10.1039/c8cp00253c

**Published:** 2018-03-29

**Authors:** Stuart J. Elliott, Benno Meier, Basile Vuichoud, Gabriele Stevanato, Lynda J. Brown, Javier Alonso-Valdesueiro, Lyndon Emsley, Sami Jannin, Malcolm H. Levitt

**Affiliations:** a School of Chemistry , University of Southampton , Southampton SO17 1BJ , UK . Email: B.Meier@soton.ac.uk ; Email: mhl@soton.ac.uk; b Université de Lyon , CNRS , Université Claude Bernard Lyon 1 , ENS de Lyon , Institut des Sciences Analytiques , UMR 5280 , 69100 Villeurbanne , France; c Institut des Sciences et Ingénierie Chimiques , Ecole Polytechnique Fédérale de Lausanne (EPFL) , Batochime , CH-1015 Lausanne , Switzerland

## Abstract

Monodeuterated methyl groups may support a long-lived nuclear spin state, with a relaxation time exceeding the conventional spin–lattice relaxation time *T*_1_. Dissolution-DNP (dynamic nuclear polarization) may be used to hyperpolarize such a long-lived spin state. This is demonstrated for the CH_2_D groups of a piperidine derivative. The polarized sample is manipulated in the ambient magnetic field of the laboratory, without destruction of the hyperpolarized singlet order. Strongly enhanced CH_2_D signals are observed more than one minute after dissolution, even in the presence of paramagnetic radicals, by which time the NMR signal from the hyperpolarized proton magnetization has completely disappeared.

## Abstract 2

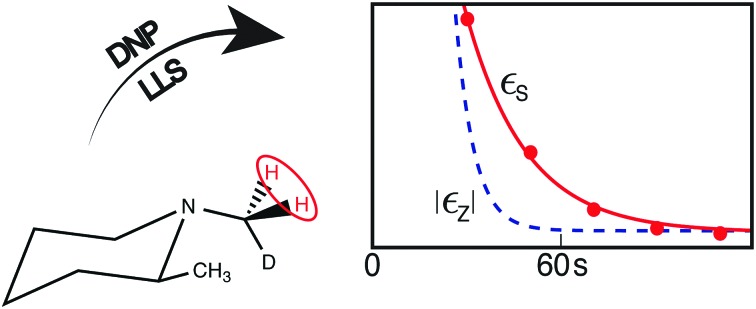
Dissolution-dynamic nuclear polarization is implemented to hyperpolarize long-lived nuclear spin states in monodeuterated methyl groups.

## 1. Introduction

Conventional nuclear magnetic resonance (NMR) experiments are limited by low sensitivity and weak signals. Hyperpolarization techniques such as dynamic nuclear polarization (DNP) enhance NMR signals by several orders of magnitude,^1^–^5^ with applications to ligand-binding, drug transport and metabolic tracing.^6^–^15^ However, applications of hyperpolarized NMR are strongly limited by the decay of polarization in solution, characterised by the spin–lattice relaxation time *T*_1_. Nuclei with a high gyromagnetic ratio tend to have short relaxation times, due to their relatively strong nuclear magnetism.

The restricted lifetime of hyperpolarized magnetization may be overcome by using long-lived states (LLS).^16^–^28^ In the case of spin-1/2 pairs, the long-lived state is termed singlet order, and is defined as the mean population imbalance between the singlet state |S_0_ and the three triplet states |T_*M*_, *M* ∈ {0, ±1}, where the singlet state is antisymmetric with respect to spin exchange, and the three triplet states are exchange-symmetric. Singlet order is protected against intra-molecular dipole–dipole relaxation and other symmetric decay mechanisms, and typically has an extended lifetime *T*_S_ > *T*_1_. Values of *T*_S_ exceeding 1 hour in room temperature solution have been reported for ^13^C spin pairs.^29^

**Fig. 1:**
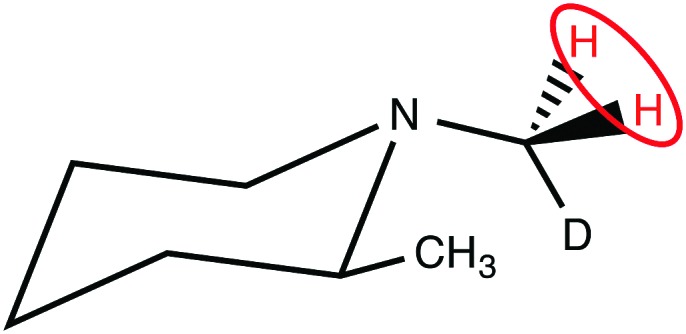
Molecular structure of (N-CH_2_D)-2-methylpiperidine (**I**). The pair of protons participating in the singlet order is circled.

The CH_2_D protons of (N-CH_2_D)-2-methylpiperidine (**I**), which have a 14 ppb chemical shift difference due to the chiral environment generated by the nearby methyl substituent (Fig. 1),^30^–^32^ have recently been shown to allow coherent access to a long-lived nuclear singlet state.^31^

The hyperpolarization of spin-pair systems leads to the generation of singlet order.^33^–^35^ DNP achieves a high nuclear Zeeman polarization *p*_Z_, which may be associated with a very low nuclear spin temperature, on the order of milliKelvin. If the spin temperature is assumed to be uniform, a nuclear singlet polarization is also generated, given by:^33^1
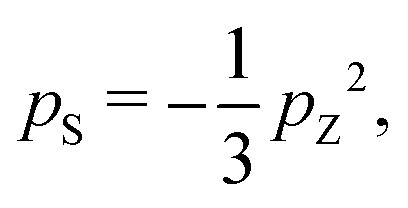
and is immediately available after dissolution.

In this communication, we hyperpolarize (N-CH_2_D)-2-methylpiperidine using DNP and demonstrate coherent readout of the long-lived spin order by applying a singlet-to-magnetization (S2M) pulse sequence.^36^,^37^ The hyperpolarized material is manipulated on the laboratory bench without destroying the hyperpolarized singlet order. The singlet order can be converted into enhanced NMR signals even when the hyperpolarized proton magnetization has completely vanished.

The direct generation of hyperpolarized singlet order by DNP was first demonstrated for the case of [1,2-^13^C_2_]pyruvic acid.^33^ However, in that case, the large chemical shift difference between the ^13^C sites caused rapid singlet decay in high magnetic field, and no significant advantage could be demonstrated over conventional Zeeman polarization. The direct generation of singlet order by DNP was also demonstrated in magnetically-equivalent systems^34^,^35^ but in these systems chemical reactions or weak cross-relaxation processes are required to generate observable signals.^34^,^35^

## 2. Experiments

### 2.1 Singlet order *vs.* magnetization

**Fig. 2:**
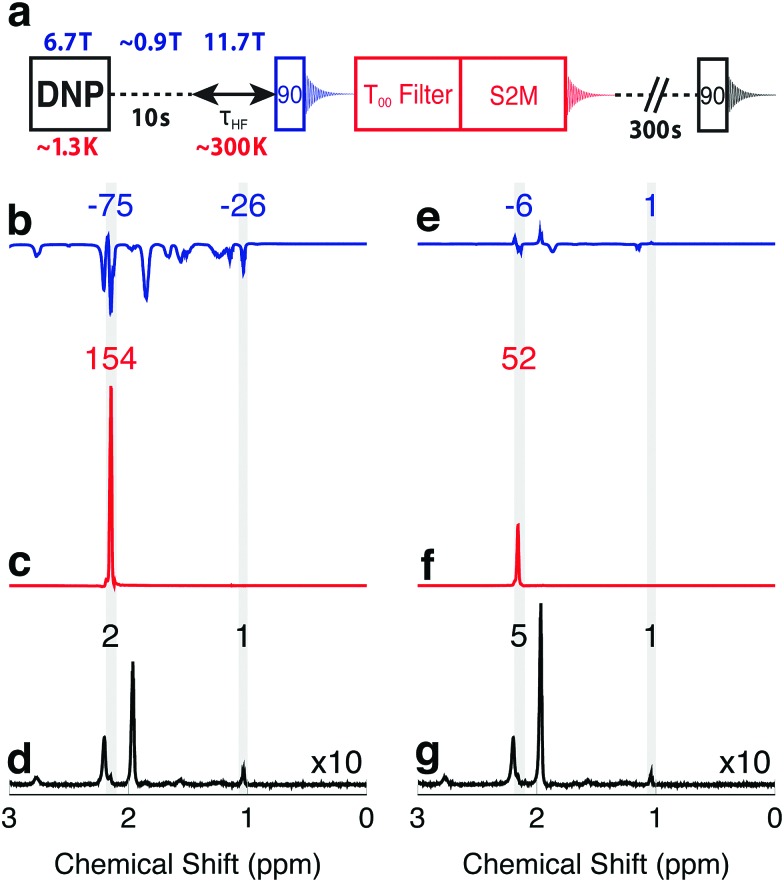
(a) Timing sequence for acquiring spectra from the hyperpolarized magnetization, the hyperpolarized singlet order, and in thermal equilibrium, from the same sample. The *T*_00_-filter and S2M sequences are described in the ESI.† (b–d) Spectra obtained from a solution of **I**, hyperpolarized in the negative sense by DNP, and with an injection and waiting interval *τ*_HF_ = 5 s after arrival in the high field magnet. (b) Spectrum from hyperpolarized magnetization showing negatively enhanced signals; (c) spectrum from singlet order converted into magnetization by the S2M sequence, showing a strongly enhanced CH_2_D signal; (d) thermal equilibrium spectrum. (e–g) Similar spectra obtained on a second hyperpolarized sample, using an injection and waiting interval *τ*_HF_ = 25 s after arrival in the high field magnet. Peak integrals are given above the spectra.

The enhanced NMR signals from hyperpolarized magnetization and singlet order in **I** are compared by using the procedure sketched in Fig. 2(a). A sample of **I** is co-mixed with 25 mM TEMPOL radical in a glass-forming solvent and polarized in the negative sense at a field of 6.7 T at a temperature of ∼1.3 K by ∼188.3 GHz frequency modulated microwave irradiation,^3^ see the ESI.† The hyperpolarized sample is dissolved in deuterated acetonitrile preheated to 410 K (pressure ∼ 10 bar) and transferred into a 11.7 T NMR magnet through a ∼0.9 T “magnetic tunnel” (transfer time ∼ 10 s).^38^ After a variable high field waiting time *τ*_HF_, a π/2 pulse is applied and the NMR signal acquired (blue in Fig. 2). Note that the single pulse and signal acquisition leaves any DNP-generated singlet order unperturbed, to a good approximation. The N-CH_2_D group singlet order is read out by applying a *T*_00_ filter sequence, followed by a S2M pulse sequence, with a combined duration of 2 s.^31^,^35^–^37^ The *T*_00_ filter quenches all NMR signals not originating from CH_2_D singlet order, and the S2M pulse sequence converts hyperpolarized singlet order into transverse magnetization, leading to a second NMR signal (red in Fig. 2). The sample is allowed to rest in the 11.7 T magnet for an additional 300 s in order to achieve thermal equilibrium, and a third NMR signal acquired using a π/2 pulse (black in Fig. 2). Fourier transformation of this signal provides the thermal equilibrium spectrum.

Fig. 2(b–d) show the spectra obtained with a delay of *τ*_HF_ = 5 s, the shortest possible delay after sample dissolution, transport and arrival in the high field magnet. Relevant spectral ranges (CH_2_D and CH_3_ group resonances) are shaded in grey, and the integrals across these ranges are given above the spectra. All integrals are normalized to the intensity of the fully protonated methyl group at 1.09 ppm in the thermal equilibrium spectrum. The signal originating from the CH_2_D group is at 2.20 ppm, and is partially obscured by a water impurity signal at 2.24 ppm. The acetonitrile solvent resonance is at 1.98 ppm.

The spectrum generated by the initial π/2 pulse is shown in Fig. 2b, and displays enhancements of –75 and –26 for the CH_2_D and CH_3_ spectral regions, respectively. These signals originate from the hyperpolarized magnetization, with the negative sign reflecting the sense of the DNP. The signal obtained from the directly hyperpolarized singlet order is shown in Fig. 2c, and clearly exceeds the signal from the hyperpolarized proton magnetization, displaying an enhancement of +154. Only the CH_2_D proton signal appears in Fig. 2c, since the *T*_00_ sequence suppresses signals from **I** which do not pass through singlet order.

The advantage of using hyperpolarized singlet order over hyperpolarized magnetization is even more pronounced at longer high field waiting times *τ*_HF_. Spectra obtained with *τ*_HF_ = 25 s are displayed in Fig. 2(e–g) and show only weak traces of signals from the hyperpolarized magnetization. The signal obtained from hyperpolarized singlet order at *τ*_HF_ = 25 s, on the other hand, still gives an enhancement of more than 50 relative to thermal equilibrium.

### 2.2 Decay of hyperpolarized singlet order

**Fig. 3:**
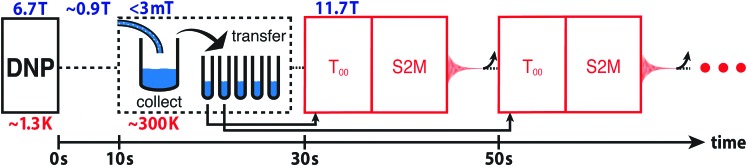
Procedure for monitoring the decay of hyperpolarized singlet order in (N-CH_2_D)-2-methylpiperidine. Hyperpolarized **I** is collected at low field (≤3 mT) in a flask preloaded with 2 mL degassed CD_3_CN solvent. The solution is pipetted (at low field) into 5 separate 0.5 mL NMR tubes. The first tube is inserted into the 11.7 T magnet, a *T*_00_ filter sequence is applied to select out NMR signals passing through CH_2_D singlet order,^36^,^37^ and the S2M pulse sequence converts the hyperpolarized proton singlet order into observable magnetization for detection.^31^,^35^ Within the following 20 s the sample is ejected and the next NMR tube is injected; this procedure is repeated for all five NMR tubes. The curved arrow after each signal acquisition represents the ejection of the NMR tube.

The hyperpolarized *T*_S_ is estimated by using the procedure sketched in Fig. 3. A hyperpolarized sample of **I** is flushed out of the cryostat using hot acetonitrile and collected in a flask preloaded with 2 mL degassed acetonitrile in the stray field of a 11.7 T NMR magnet (≤3 mT). The solution is divided into aliquots in the ambient magnetic field of the lab bench. The first 0.5 mL aliquot is loaded into an NMR tube and inserted into the 11.7 T NMR magnet. NMR signals are obtained from the hyperpolarized singlet order by applying a *T*_00_ filter sequence followed by a S2M pulse sequence.^36^,^37^ The tube is then ejected and a second tube is inserted that had been filled in the meantime. The delay between the measurements on the two tubes is 20 s. This process is repeated for a total of five tubes. See the ESI† for a video of the experimental procedure.

The signal enhancement factors in a Zeeman hyperpolarization experiment and a singlet hyperpolarization experiment are denoted by *ε*_Z_ and *ε*_S_ respectively. These are given by the spectral integrals of the CH_2_D peak relative to thermal equilibrium, *i.e. ε*_Z_ = *I*_Z_/*I*_eq_ and *ε*_S_ = *I*_S_/*I*_eq_, where *I*_Z_ and *I*_S_ are the integrals for the direct Zeeman and singlet hyperpolarization experiments, respectively. In practice, the intensity *I*_eq_ of the thermal equilibrium CH_2_D peak was estimated by multiplying the CH_3_ peak intensity by 2/3, in order to avoid complications caused by the overlap of the CH_2_D peak with a water impurity peak.

**Fig. 4:**
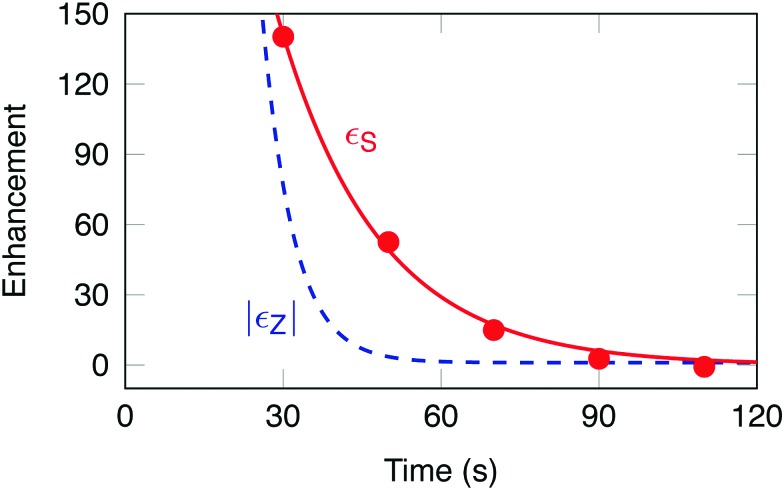
Filled circles: experimental values of the signal enhancement factor in a singlet NMR experiment *ε*_S_(*t*), as a function of the elapsed time *t* after dissolution. Solid red line: exponential decay curve given by *ε*_S_(*t*) = *ε*_S_(0) exp{–*t*/*T*_S_}, with initial enhancement *ε*_S_(0) = 680 and time constant *T*_S_ = 19.0 s. Dashed blue line: magnitude of the signal enhancement in a Zeeman polarization experiment, as inferred from the data: |*ε*_Z_(*t*)| = |*ε*_Z_(0)| exp{–*t*/*T*_1_}, with *ε*_Z_(0) = –14 750 and *T*_1_ = 5.9 s.

The experimental signal enhancement factors *ε*_S_(*t*) are shown by the filled symbols in Fig. 4. The time coordinate *t* of each point is given by the total elapsed time since dissolution, including the transport of the sample out of the polarizer, the waiting time in low field (different for each aliquot), the insertion into the high field magnet and any waiting time for stabilization before application of the pulse sequence. The data fit well to a mono-exponential decay with a time constant *T*_S_ = 19 ± 3 s, and an initial enhancement *ε*_S_(0) = 680 ± 126.

The lifetime of hyperpolarized singlet order was found to be ∼3.1 times longer than that of longitudinal magnetization, in agreement with a previous study.^31^ In prior work the chemical inequivalence at high field was suppressed by an on resonant spin-locking field, which we assume to be equivalent to storing the hyperpolarized singlet order in a ≤3 mT magnetic field. The reported singlet lifetime of 0.2 M **I** in degassed CD_3_CN solvent at 11.7 T and 25 °C is: *T*_S_ = 32.8 ± 0.6 s.^31^ Discrepancies between the reported singlet lifetimes are attributed to the presence of paramagnetic oxygen and radicals dissolved in solution.

## 3. Discussion

A direct comparison with the signal enhancement from CH_2_D Zeeman polarization is not straightforward, since the Zeeman polarization decays rapidly and the spectral analysis is complicated by peak overlap. The dashed blue curve in Fig. 4 shows an indirect estimate of *ε*_Z_(*t*) which was inferred as follows: (i) the Zeeman polarization level was estimated by comparing the DNP-enhanced solid-state NMR signal at ∼1.3 K with a thermal equilibrium signal measured at ∼4.2 K (both signals were measured in the polarizer). This comparison gave the following estimate of the Zeeman polarization level in the solid-state, prior to dissolution: *p*solidZ = –59 ± 5%, see the ESI;† (ii) it was assumed that the Zeeman polarization is substantially preserved through the dissolution process, so that *p*_Z_(0) ≃ *p*solidZ, where *p*_Z_(0) is the Zeeman polarization immediately after dissolution; (iii) the thermal equilibrium Zeeman polarization for protons in a field of 11.7 Tesla and temperature of *T* = 300 Kelvin is governed by the Boltzmann distribution, and is given by *p*eqZ = *ħγB*^0^/2*k*_B_*T* = 39.8 × 10^–6^. Combining these results gives the following best estimate for the initial signal enhancement factor in the Zeeman-polarized experiment: *ε*_Z_(0) = *p*_Z_(0)/*p*eqZ = –14 750. The dashed blue line in Fig. 4 shows the curve |*ε*_Z_(*t*)| = |*ε*_Z_(0)| exp{–*t*/*T*_1_}, where *T*_1_ = 5.9 ± 0.6 s (estimated by separate inversion-recovery experiments), see the ESI.†

Fig. 4 shows that the singlet-polarization experiment yields larger signals than the Zeeman-polarized experiment, for elapsed times of greater than ∼30 s after dissolution.

### 3.1 Singlet polarization levels

The singlet polarization *p*_S_ in the solution-state, immediately after dissolution, may be deduced from the signal enhancement factor *ε*_S_(0) through the equation:2
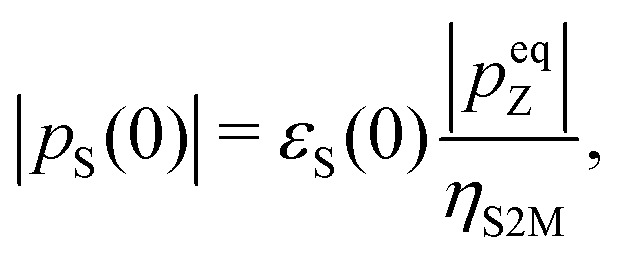
where *p*eqZ is the thermal equilibrium Zeeman polarization in high magnetic field, and *η*_S2M_ is the conversion factor for singlet order into Zeeman order using the S2M pulse sequence. As described in the ESI,† this was found experimentally to be *η*_S2M_ = 0.63 ± 0.02 for compound **I** under the relevant experimental conditions. From the thermal equilibrium Zeeman polarization *p*eqZ = 39.8 × 10^–6^ and the enhancement factor *ε*_S_(0) = 680 ± 126 (see above), we get the following estimate for the initial CH_2_D singlet polarization, immediately after dissolution: *p*_S_(0) = 4.3 ± 0.8%.

It is instructive to compare this figure with that deduced from the DNP-induced Zeeman polarization by using eqn (1). As described above, the best estimate of the Zeeman polarization level in the solid-state is *p*solidZ = –59 ± 5%. Application of eqn (1) gives the following estimate of the DNP-induced singlet polarization *p*_S_ = –12 ± 2%.

The best estimate of the CH_2_D singlet polarization, as deduced from the solid-state Zeeman polarization, is therefore ∼3 times larger than the best estimate of the same quantity measured in solution after dissolution. There are many possible reasons for this discrepancy, including the following: (i) the singlet state is an approximate eigenstate, and thermalization between the Zeeman and singlet reservoirs is incomplete at the time of dissolution, limiting the applicability of eqn (1); (ii) the violation of the high-temperature approximation may introduce spin order that is manifest neither as magnetization nor as singlet order; (iii) the concept of a uniform spin temperature under DNP may not be valid; (iv) the estimate of Zeeman polarization is associated with multiple sources of uncertainty, including the bleaching effect of radicals on the solid-state NMR signals^39^ and temperature-dependence of the detection electronics; (v) the spin dynamics during the dissolution process are not well understood, so a loss of singlet order during dissolution may not be ruled out; (vi) any possible dependence of relaxation times on magnetic field was not accounted for. Given these major sources of uncertainty, the highly qualitative agreement between the estimates of DNP-induced singlet order from the solid-state and solution-state NMR measurements is satisfactory.

## 4. Conclusion

Hyperpolarized proton singlet order may be generated directly in monodeuterated methyl groups by using a sample polarized strongly by dynamic nuclear polarization. In (N-CH_2_D)-2-methylpiperidine, the directly-generated proton singlet order is long-lived in the presence of paramagnetic radicals, and may be converted into observable magnetization by using known radiofrequency pulse techniques. The polarized sample is manipulated on the laboratory bench without destroying the hyperpolarized singlet order. We have shown that the hyperpolarized CH_2_D singlet signals may be stronger than those of the associated Zeeman polarization, since the proton singlet order decays more slowly than the CH_2_D Zeeman magnetization. These results are encouraging for future applications of hyperpolarized long-lived states.

## Conflicts of interest

There are no conflicts of interest to declare.

## Supplementary Material

Supplementary information

Supplementary movie
