# Supplementary material for: Hyperpolarized long-lived nuclear spin states in monodeuterated methyl groups
Source: Phys Chem Chem Phys. 2018 Mar 29;20(15):9755–9. doi: 10.1039/c8cp00253c (PMC5933006; doi:10.1039/c8cp00253c)
Supplement: Supplementary file 1 [file CP-020-C8CP00253C-s001.pdf]

Electronic Supplementary Material (ESI) for Physical Chemistry Chemical Physics.
This journal is © the Owner Societies 2018

Supporting Information

Hyperpolarized long-lived nuclear spin states in monodeuterated methyl groups

Stuart J. Elliott[a], Benno Meier[a], Basile Vuichoud[b], Gabriele Stevanato[c], Lynda J. Brown[a], Javier Alonso-Valdesueiro[a], Lyndon Emsley[c], Sami Jannin[b] and Malcolm H. Levitt[a]

[a] *School of Chemistry, University of Southampton, Southampton SO17 1BJ, United Kingdom*

[b] *Université de Lyon, CNRS, Université Claude Bernard Lyon 1, ENS de Lyon, Institut des Sciences Analytiques, UMR 5280, 69100 Villeurbanne, France*

[c] *Institut des Sciences et Ingénierie Chimiques, Ecole Polytechnique Fédérale de Lausanne (EPFL), Batochime, CH-1015 Lausanne, Switzerland*

Contents

1 Dissolution-dynamic nuclear polarization methods **3**

2 Solid-state polarization **4**

3 T_{00} filter and singlet to magnetization pulse sequence **5**

4 Singlet-filtered saturation recovery experiment **6**

5 Impurity **7**

6 Video of hyperpolarized long-lived state experiment **8**

7 References **9**

1. Dissolution-dynamic nuclear polarization methods

Solutions of 0.375 M (N-CH_2D)-2-methylpiperidine in the glass-forming mixture D_2O:glycerol-d_8 (50:50 v/v) were doped with 25 mM TEMPOL (purchased from Sigma-Aldrich). The synthesis of (N-CH_2D)-2-methylpiperidine is reported elsewhere [1]. The solution was sonicated for 2 minutes. Ten frozen pellets of the "DNP mixture" (10 μL volume per pellet) were inserted in the polarizer. The sample polarized at ~1.3 K and 6.7 T for ~48 minutes in a home-built polarizer by applying frequency-modulated microwave irradiation at 188.3 GHz frequency and 100 mW power [2, 3]. The microwave modulation frequency and amplitude were 10 kHz and 50 MHz, respectively. The polarized pellets were dissolved with 5 mL CD_3CN solvent (degassed via bubbling with nitrogen gas for 5 minutes) preheated to 410 K at a pressure of 10 bar. The liquid sample was transferred in 10.7 s to a 11.7 T (500 MHz) NMR magnet by pushing with helium gas at 6 bar through a PTFE tube (1.5 mm inner diameter) running inside a magnetic tunnel (0.91 T, 5 m length) [4]. 1 s was taken for sample injection and bubble dissipation. Spectra were recorded by using a 11.7 T Bruker Avance II console and processed with home-written Python software.

2. Solid-state polarization

Zeeman polarization p_Z^{solid} was accumulated in the solid-state for 0.375 M (N-CH_2D)-2-methylpiperidine in the presence of 25 mM TEMPOL radical and glassy D_2O:glycerol-d_8 matrix in a field of 6.7 T and at a temperature of ∼1.3 K under the action of negative dynamic nuclear polarization (DNP), see the dissolution-dynamic nuclear polarization methods section for more details. A Zeeman polarization of p_Z^{solid} = -59±5% was achieved in ∼48 minutes, see Fig. S1a. The solid state enhancement ϵ_Z^{solid} was approximately -360±20 compared to a spectrum recorded with the microwaves off, see Fig. S1b. The thermal spectrum was acquired after a 1 hour equilibration period at ∼4.2 K.

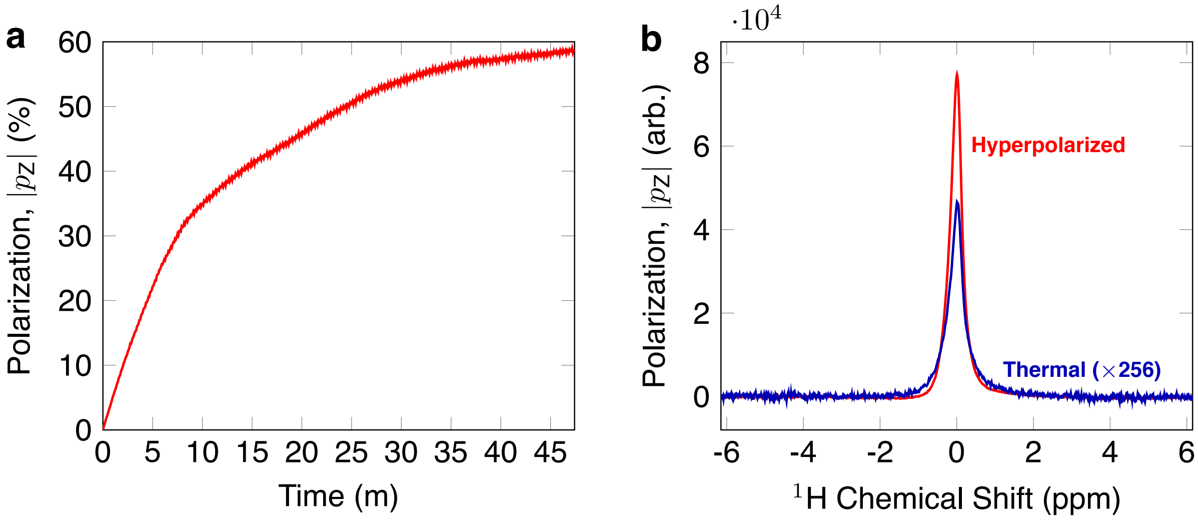

Figure S1: a) Build up of Zeeman polarization p_Z^{solid} in the solid state for 0.375 M (N-CH_2D)-2-methylpiperidine in the presence of 25 mM TEMPOL radical and glassy D_2O:glycerol-d_8 matrix in a field of 6.7 T and at a temperature of ∼1.3 K. A Zeeman polarization of p_Z^{solid} = -59±5% was reached after ∼48 minutes. b) Solid state hyperpolarized (red) and thermal (blue) spectra. The enhancement in the solid state is $\epsilon_Z^{solid} \simeq -360 \pm 20$.

3. T_{00} filter and singlet to magnetization pulse sequence

In the current study, we use the combination of a T_{00} filter and the S2M (singlet-to-magnetization) pulse sequence to retrieve singlet order generated directly from DNP. The T_{00} filter and S2M pulse sequence are shown in Fig. S2.

The singlet state is a magnetically silent arrangement of nuclear spin configurations and is unperturned by the T_{00} filter, which employs the optimized parameters shown in Table S1 to remove signals deriving from residual magnetization. Details of the T_{00} filter are also given in Refs [1, 5].

The S2M pulse sequence converts hyperpolarized singlet order into hyperpolarized transverse magnetization. The S2M pulse sequence consists of two spin-echo trains generated by a recurring sequence of a composite 180° pulse (90°_0-180°_{90}-90°_0) sandwiched between two evolution periods τ_J of duration $\frac{1}{4J}$, where J is the in pair scalar coupling (11.7 Hz). The first and second spin-echo trains are repeated n_2 and n_1 times, respectively, with $n_1 \sim n_2/2$. An additional τ_J-90°_0 module is inserted between the two echo trains [6, 7]. The parameters of the S2M pulse sequence were as follows: $\tau_J = 21.4\,\mathrm{ms}$, $n_1 = 3$ and $n_2 = 1$.

In order to determine the efficiency of the S2M sequence η_{S2M} we converted the magnetization of a thermally polarized sample to singlet order by using the M2S (magnetization "to" singlet) pulse sequence. The M2S pulse sequence is the time-reversal of the S2M pulse sequence including an initial 90°_{90}-τ_J segment. The M2S pulse sequence employs the same parameters as the S2M pulse sequence. Any remaining magnetization was quenched by using a T_{00} filter and the singlet order was back-converted to magnetization using the S2M pulse sequence. The signal was recorded and compared to a separate signal which was acquired following an excitation with a 90°_0 pulse. The ratio of the two signals was found to be 0.4. The efficiency of the S2M pulse sequence is therefore: $\eta_{S2M} = 0.4^{1/2} = 0.63 \pm 0.02$, which is close to the theoretical maximum of $\sqrt{2/3}$ [8]. The experiment was carried out on a sample of 5 μL "DNP mixture" in 0.5 mL CD_3CN solvent.

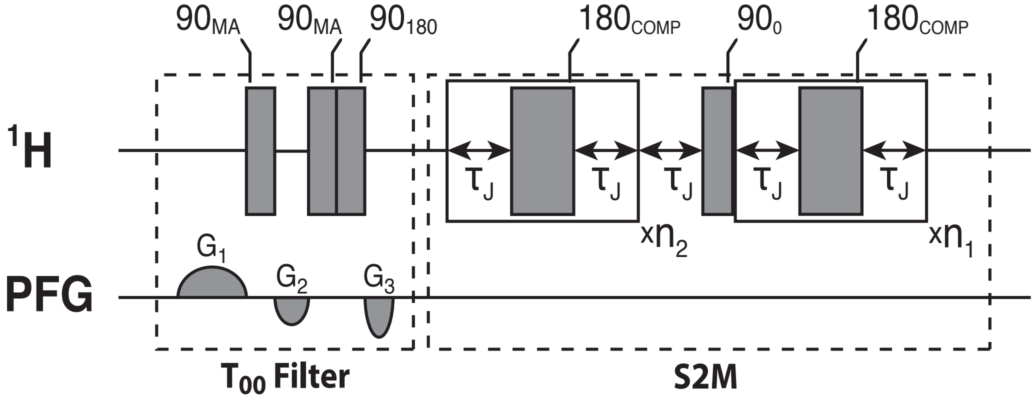

Figure S2: The T_{00} filter and S2M (singlet-to-magnetization) pulse sequence. MA denotes the magic angle (54.7°). COMP denotes a composite 180° pulse: 90°_0-180°_{90}-90°_0.

Table S1: Optimized parameters for the T_{00} filter. The T_{00} filter is implemented to suppress all signals not originating from the singlet state. NMR signals passing through spherical tensors of rank 1 or 2 are destroyed.

PFG	Shape	Strength ($\mathrm{G\,cm^{-1}}$)	Duration (ms)
G1	SINE.100	5.0	4.4
G2	SINE.100	-5.0	2.4
G3	SINE.100	-7.5	2.0

4. Singlet-filtered saturation recovery experiment

The pulse sequence for measuring the ^{1}H T_1 of the CH_2D peak obscured by the suspected water impurity is shown in Fig. S3. The scheme commences with a "saturation comb" $(90^\circ_0$ - delay$)_{100}$ which crushes all observable magnetization. The delay between 90°_0 pulses was 5 ms. After an evolution period τ_{EV}, ordinary magnetization is accrued and converted into singlet order by the M2S (magnetization to singlet) pulse sequence [6, 7]. The T_{00} filter destroys all signals not orgininating from the proton singlet order [1, 5], which is subsequently back-converted into observable magnetization by the S2M pulse sequence. The S2M applies the same transformations as the M2S but in reverse chronological order. The triplet-singlet-triplet conversion has an efficiency of 40%, see the main text for more details. NMR spectra were acquired as a function of τ_{EV} and the CH_2D T_1 of 5.9 ± 0.7 s was determined from the integrals of the resulting singlet resonances.

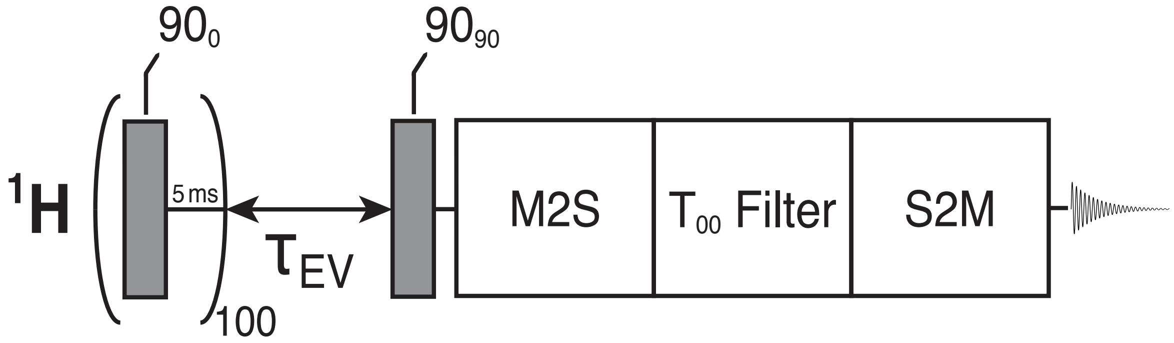

Figure S3: Pulse sequence for estimating the longitudinal relaxation time of the obscured CH_2D resonance. The "T_{00} filter" and the S2M pulse sequence are described in more detail in section 3, the main text and Refs [1-4].

5. Impurity

The resonance position of the impurity, thought to be residual water [9], was found to be dependent on the volume of DNP mixture ((N-CH_2D)-2-methylpiperidine, glass-forming D_2O:glycerol-d_8 (50:50 v/v) and TEMPOL) dissolved in CD_3CN solvent, see table S2. At higher concentrations of "DNP mixture", the water impurity was shifted sufficiently far downfield such that the CH_2D resonance was unobscured in the proton NMR spectrum. At a 10 μL volume of "DNP mixture" dissolved in CD_3CN solvent, the CH_2D peak was observed at 2.19 ppm. For "DNP mixture" concentrations $<2\,\mu$L, such as those achieved after dissolution, the CH_2D peak was obscured by a more intense water resonance. The resonance shift of the water impurity as a function of "DNP mixture" volume is approximately linear, but is not currently understood. A plausible mechanism would be an exchange interaction between the -OH protons of the TEMPOL radical with those of the residual protonated water belonging to the glassy matrix. Such an exchange interaction could simultanesouly lead to a downfield peak shift for water and broader NMR lines. We are not aware of previous reports of similar phenomena.

Table S2: Resonance position of the suspected water impurity for different volumes of (N-CH_2D)-2-methylpiperidine, glass-forming D_2O:glycerol-d_8 (50:50 v/v) and TEMPOL mixture dissolved in 0.5 mL degassed CD_3CN solvent at 11.7 T (500 MHz) and 25°C. Chemical shifts were referenced with respect to the CD_3CN solvent peak.

Volume (μL)	Position (ppm)
2	2.217
5	2.297
10	2.389

6. Video of hyperpolarized long-lived state experiment

Please see the online, electronic Supporting Information (SI) for the video of our hyperpolarized long-lived state experiment.

7. References

[1] Elliott, S.J., Brown, L.J., Dumez, J.-N. & Levitt, M.H., *Phys. Chem. Chem. Phys.* **18**, 17965–17972 (2016).

[2] Bornet, A., Ji, X., Mammoli, D., Vuichoud, B., Milani, J., Bodenhausen, G. & Jannin, S., *Chem. Phys. Lett.* **602** 63–67 (2014).

[3] Bornet, A., Pinon, A., Jhajharia, A., Baudin, M., Ji, X., Emsley, L., Bodenhausen, G., Ardenkjær-Larsen, J.-H. and Jannin, S., *Phys. Chem. Chem. Phys.* **18** 30530–30535 (2016)

[4] Milani, J., Vuichoud, B., Bornet, A., Miéville, P., Mottier, R., Jannin, S. & Bodenhausen, G., *Rev. Sci. Instrum.* **86** 024101 (2015).

[5] Mammoli D., Vuichoud, B., Bornet, A., Milani, J., Dumez, J.-N., Jannin, S. & Bodenhausen, G., *J. Phys. Chem. B* **119**, 4048–4052 (2015).

[6] Pileio, G., Carravetta, M. & Levitt, M.H., *Proc. Natl. Acad. Sci. U. S. A.* **107**, 17135–17139 (2010).

[7] Tayler, M.C.D. & Levitt, M.H., *Phys. Chem. Chem. Phys.* **13**, 5556–5560 (2011).

[8] Levitt, M.H., *J. Magn. Reson.* **262**, 91–99 (2016).

[9] Fulmer, G.R., Miller, A.J.M., Sherden, N.H., Gottlieb, H.E., Nudelman, A., Stoltz, B.M., Bercaw, J.E. & Goldberg, K.I., *Organometallics* **29**, 2716–2179 (2010).
